# Enhanced Mortality to Metastatic Bladder Cancer Cell Line MB49 in Vasoactive Intestinal Peptide Gene Knockout Mice

**DOI:** 10.3389/fendo.2017.00162

**Published:** 2017-08-07

**Authors:** Niely Mirsaidi, Matthew P. Burns, Steve A. McClain, Edward Forsyth, Jonathan Li, Brittany Dukes, David Lin, Roxanna Nahvi, Jheison Giraldo, Megan Patton, Ping Wang, Ke Lin, Edmund Miller, Timothy Ratliff, Sayyed Hamidi, Scott Crist, Ken-Ichi Takemaru, Anthony Szema

**Affiliations:** ^1^Department of Technology and Society, College of Engineering and Applied Sciences, Stony Brook University, Stony Brook, NY, United States; ^2^Three Village Allergy & Asthma, PLLC, South Setauket, NY, United States; ^3^McClain Laboratories, LLC, Smithtown, NY, United States; ^4^Department of Urology, Stony Brook University School of Medicine, Stony Brook, NY, United States; ^5^The Feinstein Institute for Medical Research, Center for Heart and Lung Research, Manhasset, NY, United States; ^6^The Elmezzi Graduate School of Molecular Medicine, Manhasset, NY, United States; ^7^Hofstra Northwell School of Medicine, Hempstead, NY, United States; ^8^Purdue University, Center for Cancer Research, West Lafayette, IN, United States; ^9^Department of Comparative Pathobiology, College of Veterinary Medicine, Purdue University, West Lafayette, IN, United States; ^10^James J. Peters Veterans Affairs Medical Center, Bronx, NY, United States; ^11^Department of Pharmacological Sciences, Stony Brook University School of Medicine, Stony Brook, NY, United States; ^12^Department of Occupational Medicine, Epidemiology, and Prevention, Hofstra Northwell School of Medicine, Hempstead, NY, United States; ^13^Northwell Health, Department of Medicine, Division of Pulmonary and Critical Care, Manhasset, NY, United States; ^14^Northwell Health, Department of Medicine, Division of Allergy and Immunology, Manhasset, NY, United States

**Keywords:** vasoactive intestinal peptide gene knockout mice, MB49 murine bladder cancer, mortality, metastases, vasoactive intestinal peptide

## Abstract

To identify if the absence of the vasoactive intestinal peptide (VIP) gene enhances susceptibility to death from metastatic bladder cancer, two strains of mice were injected with MB49 murine bladder cancer cells. The growth and spread of the cancer was measured over a period of 4 weeks in C57BL/6 mice and 5 weeks in VIP knockout (KO) mice. A Kaplan–Meier plot was constructed to compare control C57BL/6 mice and C57BL/6 mice with MB49 vs. VIP KO controls and VIP KO mice with MB49. The wild-type (WT) strain (C57BL/6) contained the VIP gene, while the other strain, VIP knockout backcrossed to C57BL/6 (VIP KO) did not and was thus unable to endogenously produce VIP. VIP KO mice had increased mortality compared to C57BL/6 mice at 4 weeks. The number of ulcers between both groups was not statistically significant. *In vitro* studies indicated that the presence VIP in high doses reduced MB49 cell growth, as well as macrophage inhibitory factor (MIF), a growth factor in bladder cancer cells. These findings support the concept that VIP may attenuate susceptibility to death from bladder cancer, and that it exerts its effect via downregulation of MIF.

## Introduction

Bladder cancer is a disease which affects approximately 600,000 patients in the United States. An estimated 74,000 cases are newly diagnosed each year; and 16,000 patients die from complications due to bladder cancer annually (1). From the time of diagnosis, there is a 77.4% 5-year survival rate (1); however, survival depends on the stage at the time of diagnosis. Regional lymph node spread at the time of diagnosis yields a 34% 5-year survival. Distant metastases at the time of diagnosis entail a 5%, 5-year survival (1). Treatment often entails a combination of surgery, radiation therapy, and chemotherapy (2).

A potential novel therapeutic agent for bladder cancer is vasoactive intestinal peptide (VIP), which is a 28-amino acid peptide that has multiple therapeutic actions, including bronchodilatory and anti-inflammatory properties (3, 4). VIP inhibits the proliferation of small-cell lung cancer *in vitro* and *in vivo* in mice (5) and has been shown to upregulate nuclear expression of p53 in mouse renal cell carcinomas (6). Loss of expression of p53, a tumor suppressor, and its analogs leads to tumor growth and can also be found in patients with bladder cancer (7, 8). The finding that VIP receptors are present in bladder carcinomas (9) lends support to the concept that we may plausibly treat bladder cancer with VIP.

With the recent availability of VIP knockout (VIP KO) mice, we hypothesized that these animals have enhanced mortality to bladder cancer. VIP KO mice lack the VIP gene and thus do not have endogenous VIP.

Using a mouse bladder cancer cell line, MB49, obtained from Timothy Ratliff (Purdue University College of Veterinary Medicine), we created a model using leg injections of the cancer cells to test whether loss of the VIP gene leads to increased mortality and/or morbidity from bladder cancer metastases, compared to control C57BL/6 mice.

We then performed *in vitro* analyses of the effect on VIP on MB49 cells. We hypothesized that VIP would decrease cell growth by decreasing the activity of macrophage inhibitory factor (MIF), a known growth factor in bladder cancer cells (10).

## Materials and Methods

### General Procedures

Using 0.1 mL isoflurane, we anesthetized the VIP KO mice (*n* = 11) at a rate of 4 L/min *via* nosecone technique. We subsequently injected anesthetized animals with 0.1 mL (1E-6/100 microliters) MB49 bladder cancer cells in the right hind leg. A control group of untreated VIP KO mice (3 mice) received 0.1 mL 0.9% saline also in the right hind leg. Similarly, we anesthetized 14 C57BL/6 mice with 0.1 mL ketamine/xylazine mixture followed by 0.1 mL (1E-6/100 microliters) MB49 bladder cancer cells in the right hind leg. An untreated group of five C57BL/6 did not receive bladder cancer cells. As approved by the IACUC, mice that expressed any signs of undue distress were euthanized immediately and counted as non-survivors. Mice that did not succumb to death prior to the end of the study were euthanized at the end of the study period.

### Animal Assessment

In both cancer-injected VIP KO and C57BL/6 mice, we measured the size of tumors, visible chest wall metastases, and ulcers using a caliper. Both groups of mice were weighed during the course of the study.

### Tissue Preparation

Necropsy included lungs, heart, leg, and bladder. Samples were formalin-fixed and embedded in paraffin. 5 µm sections were cut and stained with hematoxylin & eosin (H&E). Analysis was done with all observers blinded to the conditions.

### *In Vitro* Study

MB49 mouse bladder cancer cells were cultured in RPMI 1640 containing 10% fetal bovine serum, and 1% penicillin/streptomycin (11). The cells were seeded in 35 mm dishes at a density of 10^4^ cells per well, and cultured for 5 days at 37°C in a 5% CO_2_ atmosphere, in the presence or absence of VIP. At the end of the culture period, the adherent cells in each culture vessel were counted, and expressed as a percentage of control (no VIP). Cell culture medium was collected and assayed for the concentration of macrophage MIF using a commercially available ELISA (R&D Systems, Minneapolis, MN, USA).

### Statistical Analysis

A Kaplan–Meier curve was constructed to compare mortality rates between VIP KO and C57BL/6 mice.

## Results

### Tumor Burden Effects on Animal Weight

Over a period of 5 weeks, 6 out of 11 (55%) VIP KO mice experienced slight weight loss, and the remaining mice experienced weight gain. While the majority of this weight gain was slight, one of the VIP KO mice (9%) had a significant weight gain by comparison (approximately 13 g). Similarly, the majority of wild-type (WT) mice experienced slight decreases in weight over a period of 4 weeks. However, 3/19 (16%) WT mice experienced a slight increase in weight, and 1 mouse (5%) experienced a significant (approximately 7 grams) increase in weight and 1 mouse (5%) did not change in weight.

The mean weight of the VIP KO mice were less than those of the WT mice at the beginning of the experiments, as previously reported (12). At the time of death, there was no significant change in weight in either group, despite increase in leg girth from tumor growth (Table 1).

**Table 1:** Weight changes in wild-type (WT) and vasoactive intestinal peptide knockout (KO) mice.

	WT	KO
Average initial weight (g)	32.8	26.3
Average final weight (g)	32.4	27
Average percent change weight	−1	2.75

### Animal Activity and Survivability

Four out of 11 VIP KO mice expired by 5 weeks, including the 4/11 (36%) with leg ulcers. All C57BL/6 mice survived at 4 weeks despite the presence of a tumor in their right legs. Aside from the 5/19 (26%) C57BL/6 mice with ulcerated tumors, the majority of C57BL/6 mice did not particularly appear to be in distress during the growth of their carcinomas. Most were still mobile but walked with a limp due to the large size of the tumors.

### Tumor Progression

**Figure 1:**
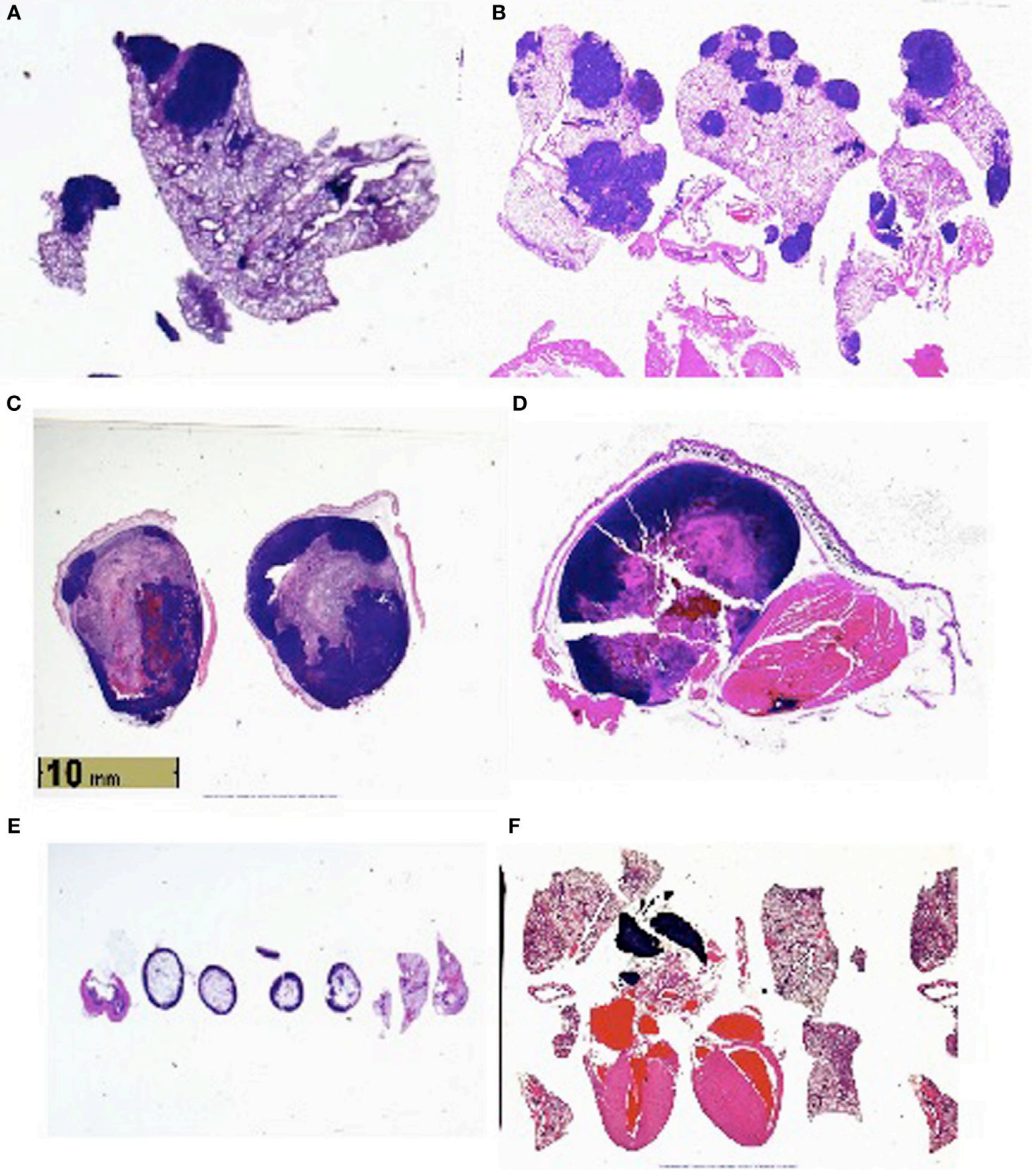
**(A)** Lung metastases in vasoactive intestinal peptide knockout (VIP KO) mouse. **(B)** Lung metastases in C57BL/6 mouse. **(C)** Leg tumor in VIP KO mouse. **(D)** Leg tumor of C57BL/6 mouse. **(E)** Histology of healthy VIP KO mouse, including lungs. **(F)** Histology of healthy C57BL/6 mouse, including heart and lungs.

Histologic examination (Figure 1) reveals metastases to the lungs, and in certain animals’ perithymic, pericardiac, intracardiac, and intravascular lesions.

### Tumor Measurement *In Vitro*

MB49 cells grown *in vitro* in the presence of 150 mg/ml VIP showed decreased cell growth when compared to cells grown in the absence of VIP (Figure 3). Compared to cells grown in the absence of VIP, those grown in the presence of VIP also showed decreased extracellular MIF accumulation (Figure 4), a molecule known to promote proliferation of bladder cancer cells (10).

## Discussion

In a previous study, we determined that loss of VIP gene led to increased mortality in association with progressive right ventricular hypertrophy (12). We additionally determined that VIP KO mice have reduced survival at 20 months (100% mortality), as opposed to 100% survival among WT C57BL/6 mice (12). In this study, VIP KO mice have a higher mortality rate with exposure to MB49 bladder cancer cell line than C57BL/6 mice, supporting the concept that VIP inhibits the susceptibility to death from bladder cancer. Additionally, other studies have shown that PAC1, VPAC1, and VPAC2 receptor transcripts were expressed in the urothelium and detrusor smooth muscle of mouse urinary bladder (13), which suggests VIP’s potential role in regulating bladder cancer cell growth. We found that administration of VIP to bladder cancer cells cultured *in vitro* led to decreased cell growth, suggesting that the presence of VIP plays a role in regulating bladder cancer cell proliferation, at least in part through VPAC1 receptor. These findings are supported by our *in vitro* data in which VIP induced a dose dependent decrease in the proliferation of the bladder cancer cell line, and reduced the elaboration of MIF, which is known to increase cancer cell proliferation and angiogenesis (10, 14, 15).

In addition, given that the MB49 and C57BL/6 models share similarities with human bladder cancer regarding cell surface markers, sensitivity to apoptosis and immunological profiles (16, 17), our findings lend plausibility to studying whether human patients with late stage bladder cancer have reduced VIP expression in serum compared to those with early stage disease.

**Figure 2:**
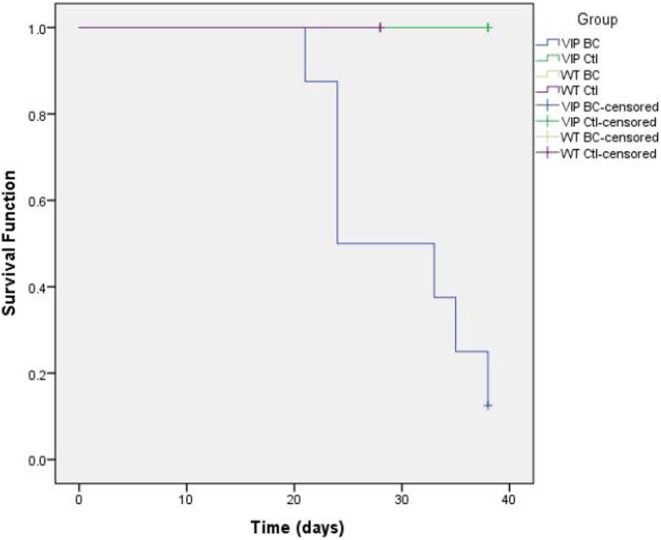
Kaplan–Meier plot comparing death rates between vasoactive intestinal peptide knockout (VIP KO) controls, VIP KO mice with MB49, C57BL/6 controls, and C57BL/6 mice with MB49 (*p* = 0.002).

Furthermore, in C57BL/6 mice, tumor growth did not seem to change gross mobility or longevity, despite histology showing the spread of cancer to the lungs, heart, vascular and lymphatic systems, and bone of several mice. The Kaplan–Meier plot (Figure 2) shows statistically significantly (*p* = 0.002) increased mortality among VIP KO mice injected with MB49 bladder cancer, whereas all other mice (VIP KO controls, WT controls, and WT mice injected with cancer) survived until the end of term. This increased mortality in VIP KO mice is not associated with an increased number of pulmonary metastases or weight loss or tumor size.

**Figure 3:**
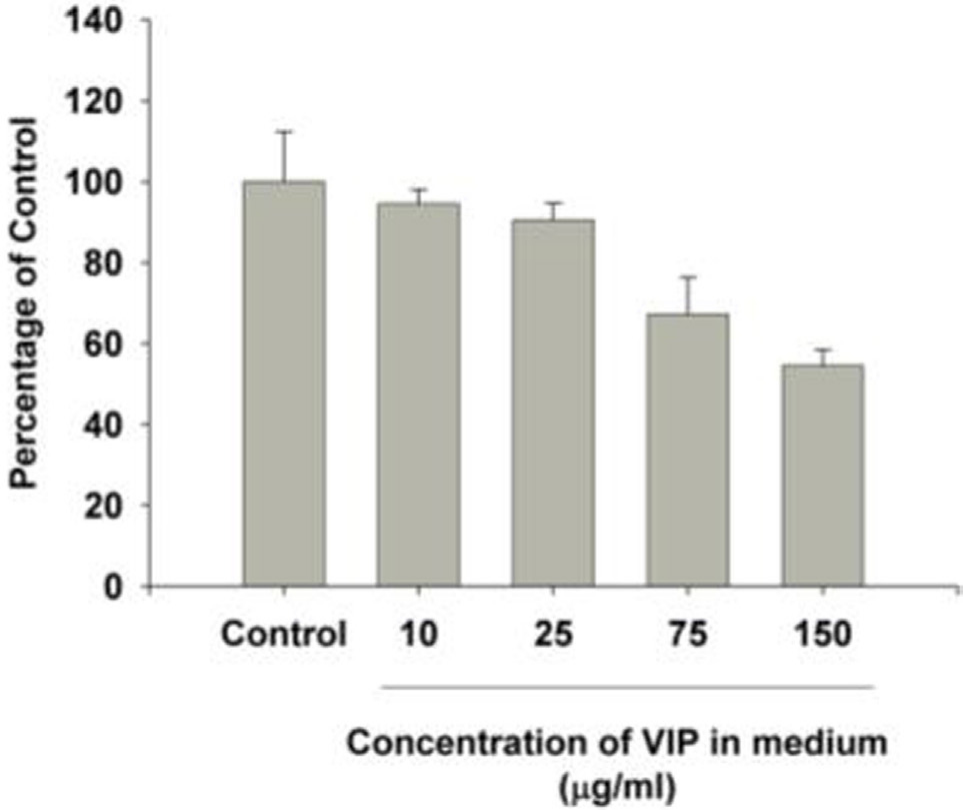
Vasoactive intestinal peptide (VIP) concentration-dependent changes to bladder cancer cell count *in vitro*. The growth in each group was significantly different from control cells grown in the absence of VIP. *p* < 0.001 [multiple comparisons vs. control group (Holm–Šidak method)].

**Figure 4:**
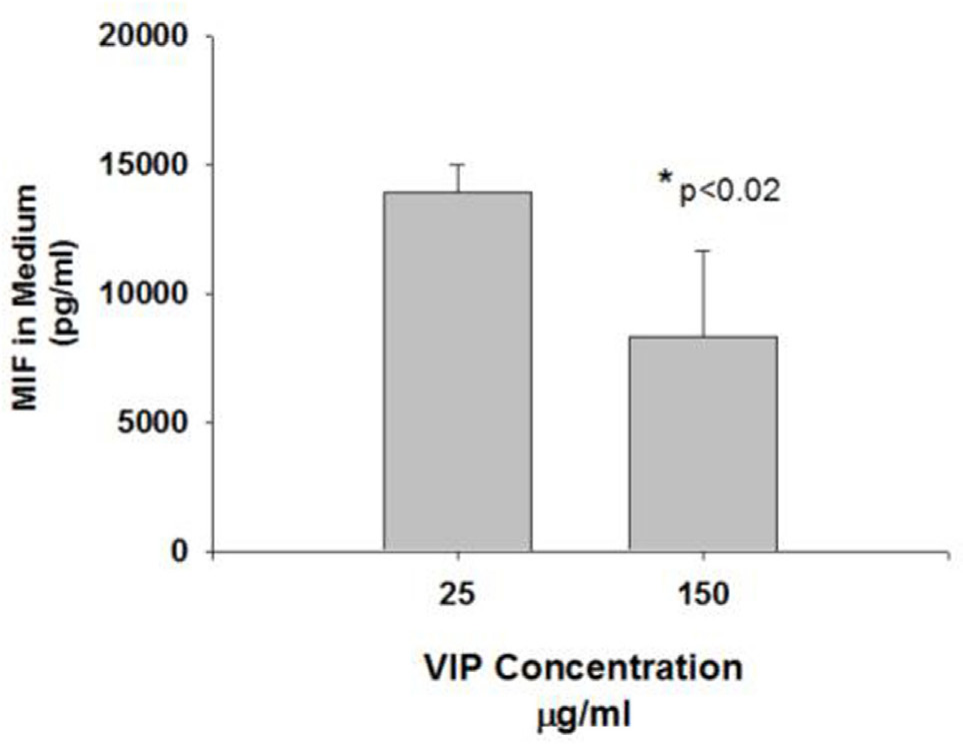
Vasoactive intestinal peptide (VIP) concentration-dependent changes to MIF in medium. The MIF concentration in the cells treated with VIP 150 mg was significantly reduced compared to controls (*p < 0.05).

## Conclusion

Vasoactive intestinal peptide KO mice have enhanced susceptibility to death from MB49 bladder cancer injected in the hind leg. Despite the growth and spread of tumors in both VIP KO and C57BL/6 groups injected with cancer cells, only VIP KO mice died prior to the end of the study. Neither VIP KO controls nor WT controls developed or succumbed to cancer. The Kaplan–Meier analysis further suggests that the lack of endogenous VIP production significantly increases the likelihood of death to cancer. Both VIP KO and C57BL/6 bladder-injected mice had metastases to the lung; however, larger and more numerous metastases were seen in C57BL/6 mice. Vascular invasion was seen in both groups.

Vasoactive intestinal peptide, which currently does not have a therapeutic indication, may potentially be a novel medicine to treat bladder cancer. Further studies into the mechanism of VIP’s action on bladder cancer are nonetheless warranted to understand the full benefit of *in vivo* VIP administration as well as any potential side effects related to dosage.

## Ethics Statement

Stony Brook University’s Institutional Animal Care and Use Committee (IACUC) approved this study.

## Author Contributions

NM and MB were responsible for injection, husbandry and surgery of C57BL/6 mice, data collection, and manuscript preparation. SM prepared slides and performed histological examination on the animal tissue. EF, JL, BD, and DL were responsible for injection and husbandry of VIP KO mice and data collection. JG was responsible for injection of C57BL/6 mice and animal surgery. RN was responsible for data interpretation and manuscript preparation. MP was responsible for injection and husbandry of C57BL/6 mice and data collection. EM, PW, and KL conducted *in vitro* studies and were responsible for data collection and revision of the manuscript. SH analyzed mortality rates between C57BL/6 mice and VIP KO mice and helped revise the manuscript. TR and SC provided MB49 bladder cancer cells. K-IT provided funding and cultured the MB49 bladder cancer cells. AS was responsible for injection, husbandry and surgery of C57BL/6 mice, data collection, manuscript preparation, and supervision of the study.

## Conflict of Interest Statement

The authors declare that the research was conducted in the absence of any commercial or financial relationships that could be construed as a potential conflict of interest.

## References

[1] Vacas E Bajo AM Schally AV Sánchez-Chapado M Prieto JC Carmena MJ. Vasoactive intestinal peptide induces oxidative stress and suppresses metastatic potential in human clear cell renal cell carcinoma. Mol Cell Endocrinol (2013) 365:212–22. 10.1016/j.mce.2012.10.021 23123564

[2] Bethesda M National Cancer Institute: PDQ^®^ Bladder Cancer Treatment. National Cancer Institute (2015). Available from: www.cancer.gov

[3] Szema AM Hamidi SA Lyubsky S Dickman KG Mathew S Abdel-Razek T Mice lacking the VIP gene show airway hyperresponsiveness and airway inflammation, partially reversible by VIP. Am J Physiol Lung Cell Mol Physiol (2006) 291:L880–6. 10.1152/ajplung.00499.2005 16782752

[4] Szema AM Hamidi SA Golightly MG Rueb TP Chen JJ VIP regulates the development & proliferation of Treg in vivo in spleen. Allergy Asthma Clin Immunol (2011) 7:19 10.1186/1710-1492-7-19 22126441 PMC3286388

[5] Maruno K Said SI VIP inhibits the proliferation of small-cell and nonsmall-cell lung carcinoma. Ann N Y Acad Sci (1996) 805:389–92; discussion 392–3. 10.1111/j.1749-6632.1996.tb17499.x 8993419

[6] Vacas E Muñoz-Moreno L Fernández-Martínez AB Bajo AM Sánchez-Chapado M Prieto JC Signalling pathways involved in antitumoral effects of VIP in human renal cell carcinoma A498 cells: VIP induction of p53 expression. Int J Biochem Cell Biol (2014) 53:295–301. 10.1016/j.biocel.2014.05.036 24905957

[7] Urist MJ Di Como CJ Lu ML Charytonowicz E Verbel D Crum CP Loss of p63 expression is associated with tumor progression in bladder cancer. Am J Pathol (2002) 161:1199–206. 10.1016/S0002-9440(10)64396-9 12368193 PMC1867279

[8] Puzio-Kuter AM Castillo-Martin M Kinkade CW Wang X Shen TH Matos T Inactivation of p53 and Pten promotes invasive bladder cancer. Genes Dev (2009) 23:675–80. 10.1101/gad.1772909 19261747 PMC2661614

[9] Reubi JC Läderach U Waser B Gebbers JO Robberecht P Laissue JA Vasoactive intestinal peptide/pituitary adenylate cyclase-activating peptide receptor subtypes in human tumors and their tissues of origin. Cancer Res (2000) 60:3105–12. 10850463

[10] Choudhary S Hegde P Pruitt JR Sielecki TM Choudhary D Scarpato K Macrophage migratory inhibitory factor promotes bladder cancer progression via increasing proliferation and angiogenesis. Carcinogenesis (2013) 34:2891–9. 10.1093/carcin/bgt239 23825153 PMC3845890

[11] Günther JH Jurczok A Wulf T Brandau S Deinert I Jocham D Optimizing syngeneic orthotopic murine bladder cancer (MB49). Cancer Res (1999) 59:2834–7. 10383142

[12] Szema AM Hamidi SA. Gene deletion of VIP leads to increased mortality associated with progressive right ventricular hypertrophy. J Cardiovasc Dis (2014) 2:131–6. 24860842 PMC4031245

[13] Girard BM Malley SE Braas KM May V Vizzard MA. PACAP/VIP and receptor characterization in micturition pathways in mice with overexpression of NGF in urothelium. J Mol Neurosci (2010) 42:378–89. 10.1007/s12031-010-9384-3 20449688 PMC2955834

[14] Shimizu T Abe R Nakamura H Ohkawara A Suzuki M Nishihira J. High expression of macrophage migration inhibitory factor in human melanoma cells and its role in tumor cell growth and angiogenesis. Biochem Biophys Res Commun (1999) 264:751–8. 10.1006/bbrc.1999.1584 10544003

[15] Nobre CC de Araújo JM Fernandes TA Cobucci RN Lanza DC Andrade VS Macrophage migration inhibitory factor (MIF): biological activities and relation with cancer. Pathol Oncol Res (2016) 23(2):235–44. 10.1007/s12253-016-0138-6 27771887

[16] Loskog A Dzojic H Vikman S Ninalga C Essand M Korsgren O Adenovirus CD40 ligand gene therapy counteracts immune escape mechanisms in the tumor microenvironment. J Immunol (2004) 172:7200–5. 10.4049/jimmunol.172.11.7200 15153545

[17] O’Donnell MA Luo Y Hunter SE Chen X Hayes LL Clinton SK. Interleukin-12 immunotherapy of murine transitional cell carcinoma of the bladder: dose dependent tumor eradication and generation of protective immunity. J Urol (2004) 171:1330–5. 10.1097/01.ju.0000109742.88380.a2 14767343

