# Supplementary material for: Enhanced Mortality to Metastatic Bladder Cancer Cell Line MB49 in Vasoactive Intestinal Peptide Gene Knockout Mice
Source: Front Endocrinol (Lausanne). 2017 Aug 7;8:162. doi: 10.3389/fendo.2017.00162 (PMC5545686; doi:10.3389/fendo.2017.00162)
Supplement: Supplementary file 1 [file Table_1.DOCX]

| **Mouse #** | **Strain** | **Control/Cancer**  **Status** | **Sex** | **Tumor Dimensions at Time of Death**  **L x W (mm)** | **Metastasis Dimensions** | **Ulcers Present?** |
| --- | --- | --- | --- | --- | --- | --- |
| 280 | VIP KO | Cancer | F | 8.6 x 8.2 | *None found* | Y |
| 281 | VIP KO | Cancer | F | 13 x 10.7 | *None found* | N |
| 282 | VIP KO | Cancer | F | 15 x 15 | 0.4 x 0.4 | Y |
| 283 | VIP KO | Cancer | F | 13 x 11 | 4 x 4 | Y |
| I | VIP KO | Cancer | F | 6 x 2 | *None found* | N |
| 285 | VIP KO | Control | F | *N/A* | *N/A* | N |
| 286 | VIP KO | Control | F | *N/A* | *N/A* | N |
| 287 | VIP KO | Control | F | *N/A* | *N/A* | N |
| 288 | VIP KO | Cancer | F | 16.7 x 10.6 | 0.35 x 0. 35 | Y |

**Supplementary Table 1(A): VIP KO Tumor Dimensions and Ulceration Information at Time of Death**

| **Mouse #** | **Strain** | **Control/Cancer Status** | **Sex** | **Tumor Dimensions**  **L x W x H (mm)** | **Lung Metastasis Dimensions**  **L x W x H (mm)** | **Ulcers Present?** |
| --- | --- | --- | --- | --- | --- | --- |
| 405 | C57BL/6 | Control | F | *N/A* | *N/A* | N |
| 406 | C57BL/6 | Control | F | *N/A* | *N/A* | N |
| 408 | C57BL/6 | Control | F | *N/A* | *N/A* | N |
| 409 | C57BL/6 | Control | F | *N/A* | *N/A* | N |
| 411 | C57BL/6 | Control | F | *N/A* | *N/A* | N |
| 401 | C57BL/6 | Cancer | M | 21 x 21 x 21 | 4 x 4 | N |
| 402 | C57BL/6 | Cancer | M | 25 x 23 x 19 | *Measurements not recorded* | N |
| 403 | C57BL/6 | Cancer | M | 11 x 4.5 x 10.9 | 4.5 x 4 | N |
| 404 | C57BL/6 | Cancer | M | 20 x 20 x 17 | 7 x 3 | Y (foot) |
| 418 | C57BL/6 | Cancer | F | 25.5 x 25.6 x 21 | 5 x 5 | N |
| 419 | C57BL/6 | Cancer | F | 11.4 x 14 x 9 | 4.5 x 2.6 | N |
| 420 | C57BL/6 | Cancer | F | 4 x 4 x 4 | *N/A* | N |
| 415 | C57BL/6 | Cancer | M | 18.1 x 12.9 x 5 | 1.5 x 3 | N |
| 416 | C57BL/6 | Cancer | M | 23 x 22 x 15 | *Measurements not recorded* | N |
| 417 | C57BL/6 | Cancer | M | 9.9 x 21 x 16 | *Measurements not recorded* | N |
| 410 | C57BL/6 | Cancer | F | 14.8 x 7.6 | *Measurements not recorded* | Y |
| 411 (CA) | C57BL/6 | Cancer | F | 17.2 x 14 x 4.8 | 0.25 x 0.25 | Y |
| 412 | C57BL/6 | Cancer | F | 27.5 x 22 x 22 | 2.2 x 1.6 | Y |
| 413 | C57BL/6 | Cancer | F | 11.4 x 8.5 x 12 | 0.15 x 0.15 (subpleural) | Y |

**Supplementary Table 1(B): C57BL/6 Tumor Dimensions and Ulceration Information at Time of Death**
