# Supplementary material for: Enhanced Mortality to Metastatic Bladder Cancer Cell Line MB49 in Vasoactive Intestinal Peptide Gene Knockout Mice
Source: Front Endocrinol (Lausanne). 2017 Aug 7;8:162. doi: 10.3389/fendo.2017.00162 (PMC5545686; doi:10.3389/fendo.2017.00162)

Supplementary Figure 1(A): Ulcerated and necrotic leg from VIP KO mouse

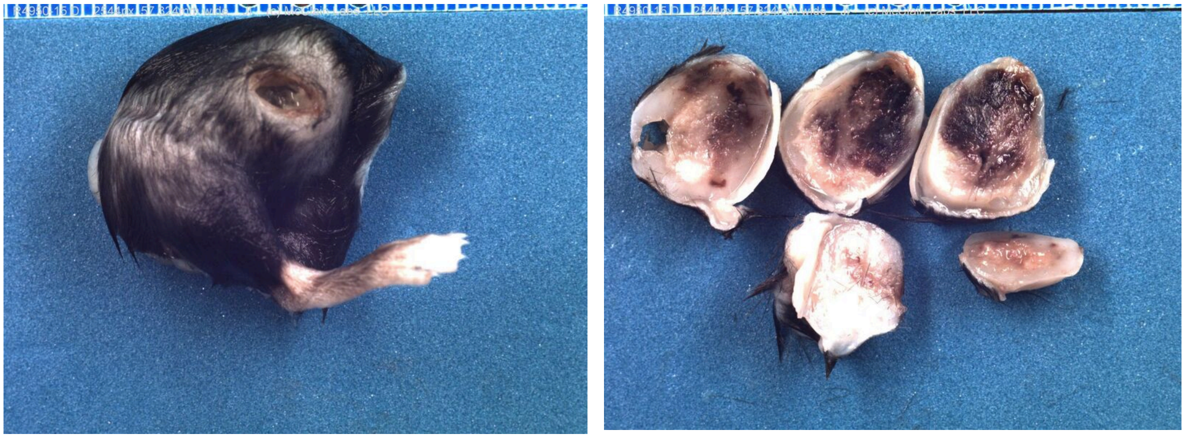

Supplementary Figure 1(B): Tumor growth in leg of C57BL/6 mouse

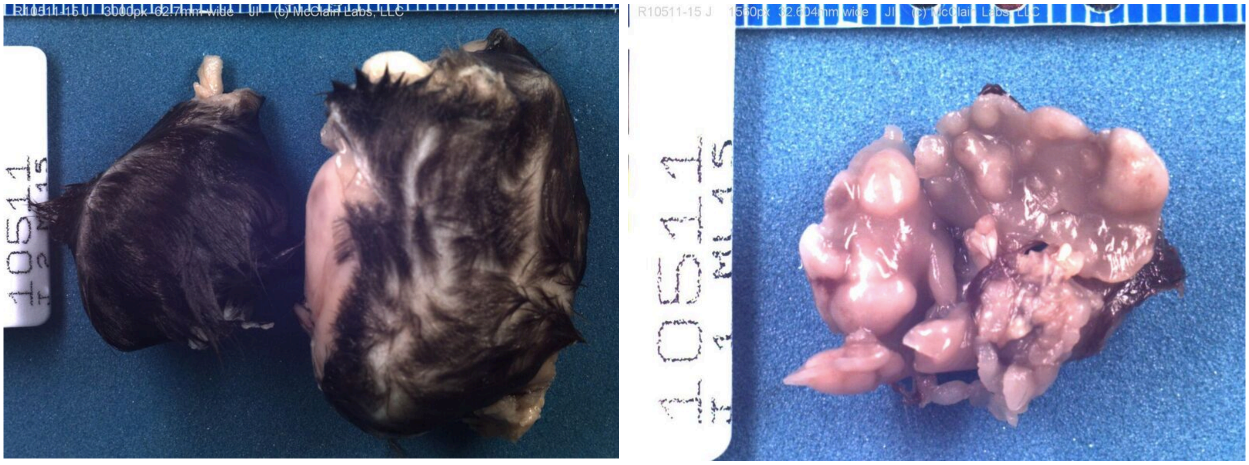

Supplement: Supplementary file 2 [file Image_1.PDF]
