# Supplementary figures and images for: Enhanced Mortality to Metastatic Bladder Cancer Cell Line MB49 in Vasoactive Intestinal Peptide Gene Knockout Mice
Source: Front Endocrinol (Lausanne). 2017 Aug 7;8:162. doi: 10.3389/fendo.2017.00162 (PMC5545686; doi:10.3389/fendo.2017.00162)

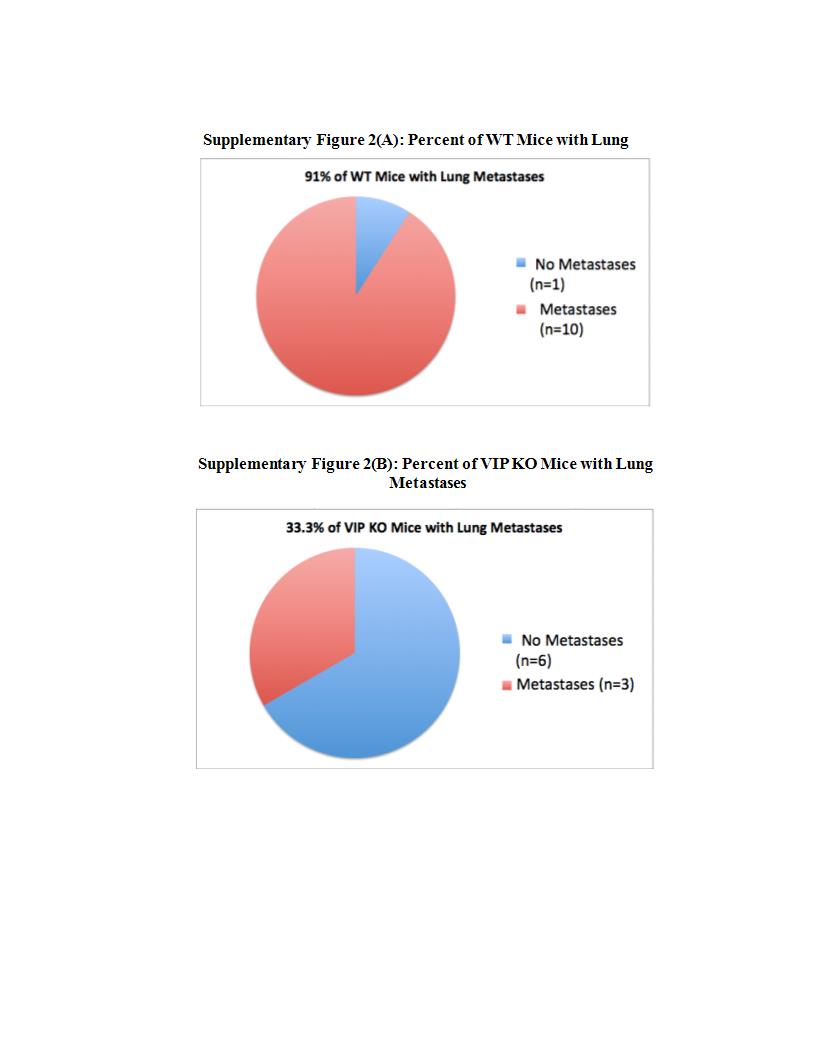

Supplement: Supplementary file 3 [file Image_2.JPEG]
